# Changes in Smoking Status and Mortality From All Causes and Lung Cancer: A Longitudinal Analysis of a Population-based Study in Japan

**DOI:** 10.2188/jea.JE20170112

**Published:** 2019-01-05

**Authors:** Ling Zha, Tomotaka Sobue, Tetsuhisa Kitamura, Yuri Kitamura, Norie Sawada, Motoki Iwasaki, Shizuka Sasazuki, Taiki Yamaji, Taichi Shimazu, Shoichiro Tsugane

**Affiliations:** 1 Division of Environmental Medicine and Population Sciences, Department of Social and Environmental Medicine, Graduate School of Medicine, Osaka University, Osaka, Japan; 2 Epidemiology and Prevention Group, Research Center for Cancer Prevention and Screening, National Cancer Center, Tokyo, Japan

**Keywords:** changes in smoking status, cohort study, mortality, reasons for smoking cessation

## Abstract

**Background:**

To update the findings of relative risk associated with smoking for all-cause mortality and that for lung cancer by considering longitudinal changes in smoking status during follow-up.

**Methods:**

Data from the JPHC study of 98,747 middle-aged Japanese adults, which started in 1990–1993, were analyzed. The information on smoking status was obtained from three questionnaire surveys (baseline, the 5th year, and the 10th year after the start of follow-up). A Poisson regression model was used to investigate the impact of smoking on mortality from all causes and lung cancer using two approaches. Model 1 used information only from baseline, while model 2 used the updated smoking status from all three surveys.

**Results:**

During the 15-year follow-up, 10,702 all-cause deaths (including 870 lung cancer cases) were identified. We compared the results obtained from two models. The relative risks associated with former smokers versus never smokers were 1.42 (95% confidence interval [CI], 1.31–1.54) among men and 1.46 (95% CI, 1.23–1.73) among women for all-cause mortality and 2.98 (95% CI, 2.09–4.24) among men and 1.83 (95% CI, 0.92–3.64) among women for lung cancer mortality, as determined using model 2. All of these were higher than the relative risks obtained from model 1. In addition, former smokers who had quit smoking due to disease during follow-up had a higher mortality risk than continuous smokers did in this study.

**Conclusions:**

The relative risks of all-cause mortality and mortality due to lung cancer among former smokers be higher than previously documented based on updated smoking status data from repeated surveys.

## INTRODUCTION

Smoking, mainly of cigarettes, is a well-established preventable risk factor for death and non-communicable diseases worldwide.^1^^–^^3^ Several reviews have evaluated studies of smoking-associated mortality and the benefits of smoking cessation.^4^^–^^7^ Improving lifestyle factors, such as smoking habits, reduces disease risk, but empirical analyses supporting these associations are currently lacking.

Although the hazard associated with smoking is substantial, general cohort studies tend to underestimate the actual death rates because they do not account for changes in smoking status; smoking information is usually acquired only once at baseline.^8^^–^^12^ Smoking habits that change during follow-up, especially when current smokers quit, affect the apparent impact of cessation. Although such problems can be reduced using repeat surveys in cohort studies,^13^ a comparison of risk estimations obtained from using updated or static data for individuals has not yet been extensively investigated.^7^^,^^14^^,^^15^

Here, we evaluated smoking status from multiple surveys during follow-up. Therefore, we could reclassify smoking status according to current information. To update findings for smoking-associated mortality, we report the relative risks for mortality according to smoking status changes.

## METHODS

### Study population

The Japan Public Health Center-based prospective study on cancer and cardiovascular diseases (JPHC Study) is a population-based cohort of 140,420 adults with registered addresses in one of 30 administrative districts supervised by 11 public health center areas. There are five public health center areas in the first group (Cohort I), which started January 1, 1990, and six in the second group (Cohort II), which started January 1, 1993.^16^ Cohort I comprised all residents aged 40–59 years at baseline, except for those in Tokyo, and Cohort II comprised all residents aged 40–69 years at baseline, except for those in Suita.^16^ Since participants from Tokyo and Suita public health centers were only evaluated at 40 or 50 years of age, 16,844 residents from these age-biased cohort areas were excluded from this analysis. After study initiation, subjects deemed ineligible were excluded, including those of non-Japanese nationality (*n* = 51), with late reports of migration occurring before the start of follow-up (*n* = 174), with incorrect birth data (*n* = 4), who refused to answer questions (*n* = 12), with duplicate registrations (*n* = 4), and who refused mail contact (*n* = 530). This study was approved by the Institutional Review Board of the National Cancer Center, Japan (Approval number: 2001-013, 14-038). Written informed consent was obtained from all the participants in the JPHC study.

### Baseline and additional surveys

Self-administered questionnaires evaluating socio-demographic characteristics, personal medical history, smoking and drinking history, diet, and other lifestyle-related factors were distributed to all registered residents at baseline (called Q00).^16^ After excluding 23,432 subjects with no baseline response, 565 without available smoking status, and 57 lost to follow-up, 98,747 individuals (47,044 men and 51,703 women) were included in this analysis. A follow-up survey was conducted during the 5th year (Q05) after baseline to evaluate any changes in lifestyle and obtain information on disease incidence during the first 5 years after the study started (called Period 1).^16^ In the 10th year after baseline (Period 2), another follow-up survey (Q10) was conducted.^16^

### Follow-up and outcome

Since all surveys were conducted at regular 5-year intervals, we defined the same follow-up duration after every survey. Thus, the follow-up period defined for participants began on January 1, 1990 and ended on or before December 31, 2004 in Cohort I and began on January 1, 1993 and ended on or before December 31, 2007 in Cohort II, so the longest follow-up duration was up to 15 years. Subjects were followed for residence status and vital status through the residential registry.^16^ Cause of death was confirmed with permission from death certificates and defined according to the 10th revision of the International Classification of Disease. The principal outcomes of this study were all-cause mortality and that due to lung cancer (International Classification of Disease code: C34).

### Exposure definition

**Figure 1.**
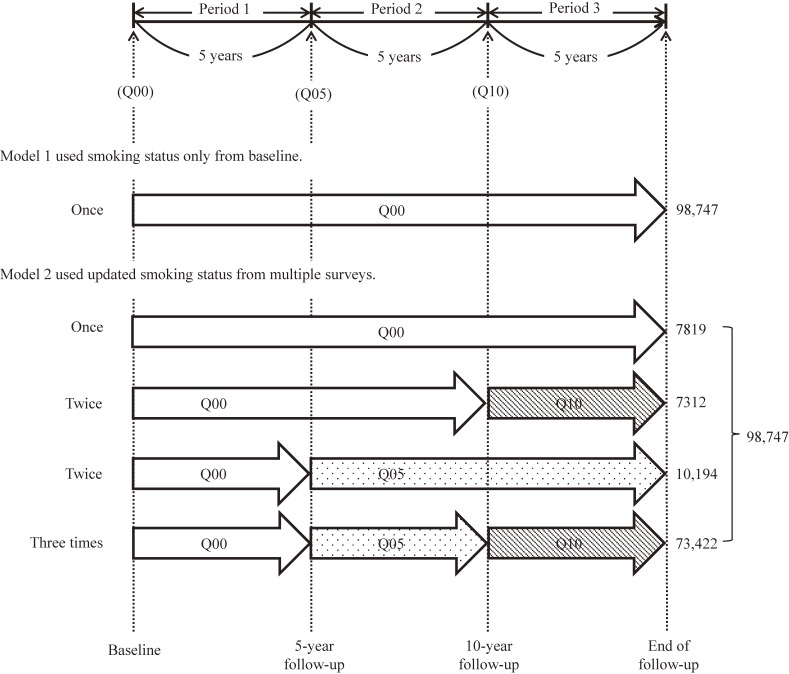
The timeline of exposure

Smoking status was initially classified as never smoker, former smoker, or current smoker. All study participants, including responders and non-responders to resurveys (Q05 and Q10), were followed for mortality. We used data on smoking status that were collected only once at Q00 in model 1, whereas in model 2 we used updated information about smoking status when available in repeat questionnaires from all three surveys (Q00, Q05, and Q10). Figure 1 shows the exposure differences on smoking status between models 1 and 2. We also defined that the information on smoking status was not changed if the individual did not answer the follow-up surveys.

In model 2, there were nine possible combinations of smoking status changes in Period 1 from Q00 to Q05 and in Period 2 from Q05 to Q10 (never smoker→never smoker, never smoker→former smoker, never smoker→current smoker, former smoker→never smoker, former smoker→former smoker, former smoker→current smoker, current smoker→never smoker, current smoker→former smoker, current smoker→current smoker). Those indicating that they were never smokers on follow-up surveys but who indicated that they were former or current smokers once before were defined as former smokers (*n* = 7,136; 7.2% of the sample).

### Reasons for quitting smoking

In the JPHC study, reasons for quitting smoking were asked at both Q05 and Q10.^16^ There were five possible responses: “Due to illness”, “Considered unhealthy for the future”, “Considered displeasing to surrounding people”, “Economic reasons”, and “Others”. Here, we divided reasons for quitting smoking into two categories based on whether participants quit smoking due to illness or not.

### Statistical methods

Statistical analyses were performed using nonparametric tests for trends and using the Student *t*-test for continuous variables. For analysis of mortality, individuals contributed to the person-years at risk during the 15 years of follow-up, and they were censored on the earliest date from death, moving out of the study areas, or the end of follow-up. Person-years, number of deaths, and relative risks and their 95% confidence intervals (CIs) for all-cause and lung cancer-related mortality were aggregated and stratified according to sex and smoking status. The analysis was conducted by Poisson regression models in models 1 and 2 separately and was adjusted by age in 5-year strata, public health center area, alcohol consumption, body mass index, medication, history of hypertension, history of diabetes, leisure-time sports, coffee consumption, and green tea consumption, which were collected at baseline.^11^^,^^12^

Life-threatening illness can cause smokers to quit, distorting death rates among current and former smokers.^5^ Therefore, we analyzed the impact of smoking status changes, focusing particularly on the reasons for quitting. We restricted sub-analyses to a 5-year follow-up from Q05 (Period 2) and another 5-year follow-up from Q10 until the study’s end (Period 3) in order to estimate the relative risks of all-cause and lung cancer-specific mortality in different smoking change combinations, especially among never smoker→never smoker, former smoker→former smoker, current smoker→former smoker, and current smoker→current smoker. In the sub-analyses, smoking status was classified by the information collected at Q05 or Q10, respectively.

Significance tests were two-sided, and we defined a *P*-value <0.05 as being statistically significant. All analyses were performed with Stata software (version 13.1; Stata Corp, College Station, TX, USA).

## RESULTS

### Changes in smoking status during the study period

Smoking statuses of participants at Q00, Q05, and Q10 are noted in Table 1. The overall proportion of current smokers during these 10 years decreased from 52.1% to 39.0% in men and from 6.1% to 4.3% in women. Accordingly, the proportion of former smokers increased from 23.9% to 39.0% in men and from 1.7% to 4.1% in women.

**Table 1.** Survey respondents on smoking status by survey period and sex

	Baseline		5-year follow-up		10-year follow-up	
		
	Number of participants	(%)	Number of participants	(%)	Number of participants	(%)
*Men*						
Responder	47,044	(100)	38,636	(100)	36,951	(100)
Never smoker	11,290	(24.0)	8,797	(22.8)	8,159	(22.1)
Former smoker	11,257	(23.9)	12,143	(31.4)	14,395	(39.0)
Current smoker	24,497	(52.1)	17,696	(45.8)	14,397	(39.0)
Non-responder	0		6,670		5,952	
Total	47,044		45,306		42,903	
*Women*						
Responder	51,703	(100)	44,980	(100)	43,783	(100)
Never smoker	47,649	(92.2)	41,352	(91.9)	40,102	(91.6)
Former smoker	882	(1.7)	1,348	(3.0)	1,795	(4.1)
Current smoker	3,172	(6.1)	2,280	(5.1)	1,886	(4.3)
Non-responder	0		5,881		5,775	
Total	51,703		50,861		49,558	

### History and cigarettes smoked daily by smoking status

Table 2 presents the information on medical history and daily cigarette consumption. Overall, 2,950 men and 587 women during Period 1 and 2,853 men and 525 women during Period 2 reported quitting smoking during the two adjacent surveys. There was a great number of participants reporting a medical history of any serious diseases, such as cancer, diabetes mellitus, stroke, ischemic heart disease, and hepatitis or cirrhosis. Those new quitters (current smoker→former smoker) who quit smoking during Period 1 (19.3%) and Period 2 (27.0%) in men were highest among all subgroups. Similarly, among both persistent quitters (former smoker→former smoker: 12.2% in Period 1 and 19.0% in Period 2) and new quitters (current smoker→former smoker: 12.1% in Period 1 and 16.4% in Period 2), the proportion of women with a medical history was significantly higher than for continuous smokers (current smoker→current smoker: 7.6% in Period 1 and 11.5% in Period 2). Since the reason for quitting smoking was asked at both Q05 and Q10 for former smokers, we calculated the proportion of new quitters (current smoker→former smoker) who quit smoking due to illness. The illnesses included some mild diseases, which were not pointed out in details in questionnaires, as well as the serious diseases mentioned above.

**Table 2.** Information on medical history and daily cigarette consumption

Sex/Changes in smoking status		Number of participants	Participants with medical history^c,d^ (%)		Number of cigarettes /day^e^	New quitters due to illness (%)	
*Men*							
Period 1 (Q00→Q05)^a^	NS→NS	8,797	949	(10.8)	—		
	FS→FS	8,787	1,298	(14.8)	16 (19.8)		
	CS→FS	2,950	570	(19.3)	20 (38.4)	893	(30.3)
	CS→CS	16,870	1,808	(10.7)	22 (13.3)		
Period 2 (Q05→Q10)^b^	NS→NS	7,314	1,111	(15.2)	—		
	FS→FS	9,773	2,056	(21.0)	23 (12.5)		
	CS→FS	2,853	770	(27.0)	24 (11.8)	1,314	(46.1)
	CS→CS	12,125	1,684	(13.9)	22 (10.3)		
*Women*							
Period 1 (Q00→Q05)^a^	NS→NS	41,352	2,851	(6.9)	—		
	FS→FS	650	79	(12.2)	9 (8.8)		
	CS→FS	587	71	(12.1)	12 (9.8)	58	(9.9)
	CS→CS	1,811	138	(7.6)	16 (9.8)		
Period 2 (Q05→Q10)^b^	NS→NS	37,049	4,234	(11.4)	—		
	FS→FS	969	184	(19.0)	14 (9.1)		
	CS→FS	525	86	(16.4)	15 (8.6)	73	(13.9)
	CS→CS	1,333	153	(11.5)	15 (7.9)		

CS, current smoker; FS, former smoker; NS, never smoker.

^a^Changes of smoking status from baseline survey to 5-year follow-up survey.

^b^Changes of smoking status from 5-year follow-up survey to 10-year follow-up survey.

^c^Diseases of medical history refers to a history of any following ones: cancer, diabetes mellitus, stroke, ischemic heart disease, and hepatitis or cirrhosis.

^d^The definition of history means the recent five years in 5-year follow-up survey, while it means all the past until Q10 in 10-year follow-up survey.

^e^Mean (standard deviation).

Table 2 also shows the mean number of cigarettes smoked daily. The means for persistent quitters (16 in men and 9 in women) and new quitters (20 in men and 12 in women) were lower than those for continuous smokers (22 in men and 16 in women) during Period 1 for both sexes. We therefore hypothesized that light smokers tended to quit smoking. However, there were no differences between former and continuous smokers during Period 2.

Since medical history refers to the most recent 5 years in the 5-year follow-up survey but refers to all the past until Q10 in 10-year follow-up survey, the percentage from Period 2 is higher compared to Period 1 because of the different definition of history.

### Relative risk by smoking status

There were 7,039 deaths in men and 3,663 deaths in women followed over a mean of 14.2 years (653,687 person-years for men and 747,526 person-years for women) documented during the study period. Results from the two approaches are shown in Table 3. In model 1, data from individuals were divided by attended age and classified only once by the Q00 smoking status. In model 2, data for each individual were divided by attended age and the three periods; smoking status was therefore reclassified based on current information. Thus, although the total number of person-years, number of deaths, and crude mortality were the same, the distribution of those in each smoking category was different between the two approaches. Compared with never smokers, the relative risks for all-cause death among current smokers were 1.79 (95% CI, 1.66–1.92) for men and 1.93 (95% CI, 1.70–2.19) for women in model 1 and 1.74 (95% CI, 1.61–1.89) for men and 1.91 (95% CI, 1.67–2.19) for women in model 2. Comparing former smokers to never smokers, the relative risks from model 2 were 1.42 (95% CI, 1.31–1.54) for men and 1.46 (95% CI, 1.23–1.73) for women, and both relative risks were greater than those from model 1 (1.22; 95% CI, 1.12–1.32 for men and 1.43; 95% CI, 1.13–1.81 for women).

**Table 3.** Comparison of all-cause and lung cancer mortality risk by smoking status between two models during the 15-year follow-up

Sex/smoking status		Number of participants (%)		Person-years (%)		All-cause					Lung cancer				
	
						Number of deaths (%)		Crude mortality	Relative risk^c^	(95% CI)	Number of deaths (%)		Crude mortality	Relative risk^c^	(95% CI)
*Men*															
Model 1^a^	Never smoker	11,290	(24.0)	159,712	(24.4)	1,190	(16.9)	745	Reference	—	51	(7.5)	32	Reference	—
	Former smoker	11,257	(23.9)	155,830	(23.8)	1,728	(24.5)	1,109	1.22	(1.12–1.32)	122	(18.0)	78	1.90	(1.34–2.72)
	Current smoker	24,497	(52.1)	338,145	(51.7)	4,121	(58.5)	1,219	1.79	(1.66–1.92)	504	(74.4)	149	4.70	(3.43–6.44)
	Total	47,044	(100)	653,687	(100)	7,039	(100)	1,077	—	—	677	(100)	104	—	—
Model 2^b^	Never smoker	—	—	151,642	(23.2)	1,073	(15.2)	708	Reference	—	42	(6.2)	28	Reference	—
	Former smoker	—	—	194,948	(29.8)	2,551	(36.2)	1,309	1.42	(1.31–1.54)	228	(33.7)	117	2.98	(2.09–4.24)
	Current smoker	—	—	307,097	(47.0)	3,415	(48.5)	1,112	1.74	(1.61–1.89)	407	(60.1)	133	4.69	(3.32–6.62)
	Total	—	—	653,687	(100)	7,039	(100)	1,077	—	—	677	(100)	104	—	—
*Women*															
Model 1^a^	Never smoker	47,649	(92.2)	690,072	(92.3)	3,214	(87.7)	466	Reference	—	155	(80.3)	22	Reference	—
	Former smoker	882	(1.7)	12,563	(1.7)	92	(2.5)	732	1.43	(1.13–1.81)	3	(1.6)	24	0.77	(0.19–3.13)
	Current smoker	3,172	(6.1)	44,891	(6.0)	357	(9.7)	795	1.93	(1.70–2.19)	35	(18.1)	78	3.28	(2.12–5.05)
	Total	51,703	(100)	747,526	(100)	3,663	(100)	490	—	—	193	(100)	26	—	—
Model 2^b^	Never smoker	—	—	683,568	(91.4)	3,176	(86.7)	465	Reference	—	152	(78.8)	22	Reference	—
	Former smoker	—	—	21,504	(2.9)	173	(4.7)	804	1.46	(1.23–1.73)	10	(5.2)	47	1.83	(0.92–3.64)
	Current smoker	—	—	42,453	(5.7)	314	(8.6)	740	1.91	(1.67–2.19)	31	(16.1)	73	3.21	(2.03–5.07)
	Total	—	—	747,526	(100)	3,663	(100)	490	—	—	193	(100)	26	—	—

CI, confidence interval.

^a^Model 1 used information on smoking status only from baseline.

^b^Model 2 used updated smoking status from all three surveys.

^c^Adjusted for age, area, alcohol consumption, body mass index, medication, history of hypertension, history of diabetes, leisure-time sports, coffee consumption, and green tea consumption.

As shown in Table 3, there were 677 deaths among men and 193 deaths among women from lung cancer. Compared with never smokers, the relative risks for lung cancer-related mortality among current smokers were 4.70 (95% CI, 3.43–6.44) for men and 3.28 (95% CI, 2.12–5.05) for women in model 1 and 4.69 (95% CI, 3.32–6.62) for men and 3.21 (95% CI, 2.03–5.07) for women in model 2. The relative risks among former smokers were 2.98 (95% CI, 2.09–4.24) for men and 1.83 (95% CI, 0.92–3.64) for women in model 2; both were greater than those from model 1 (1.90; 95% CI, 1.34–2.72 for men and 0.77; 95% CI, 0.19–3.13 for women).

### Relative risks by reasons for quitting smoking

Table 4 shows that 3,537 participants quit smoking during Period 1 and that 2,318 participants (951 because of disease and 1,367 for other reasons) supplied the reasons for smoking cessation at Q05. The relative risks for all-cause mortality among participants who quit smoking due to illness compared to never smokers was 3.54 (95% CI, 2.87–4.36), higher than that for participants who continued smoking (1.78; 95% CI, 1.57–2.00). The relative risk for lung cancer-related deaths among new quitters who quit due to illness was 6.55 (95% CI, 3.44–12.47), higher than that for continuous smokers (3.36; 95% CI, 2.19–5.15). Furthermore, of 3,378 participants who quit smoking at Q10, 2,713 reported the reasons for quitting (1,387 for disease and 1,326 for other reasons). The relative risks among new quitters due to illness were the greatest for both all-cause mortality (2.95; 95% CI, 2.48–3.50) and lung cancer-related deaths (7.90; 95% CI, 4.55–13.73).

**Table 4.** Number of deaths from all-cause and lung cancer and relative risk by changes in smoking status and reasons for quitting smoking

Follow-up period/Changes in smoking status		Number of participants	Person-years	All-cause				Lung cancer			
	
				Number of deaths	Crude mortality	Relative risk^f^	(95% CI)	Number of deaths	Crude mortality	Relative risk^f^	(95% CI)
*Period 2*^a^											
NS→NS		50,149	246,874	1,055	427	Reference	—	58	23	Reference	—
FS→FS		9,437	45,765	450	983	1.34	(1.17–1.54)	41	90	1.77	(1.08–2.90)
CS→FS	All^c^	3,537	16,880	257	1,522	2.40	(2.05–2.80)	39	231	5.45	(3.34–8.90)
	Illness^d^	951	4,383	119	2,715	3.54	(2.87–4.36)	15	342	6.55	(3.44–12.47)
	Others^e^	1,367	6,616	63	952	1.53	(1.17–2.00)	16	242	5.97	(3.18–11.22)
CS→CS		18,681	90,825	899	990	1.78	(1.57–2.00)	109	120	3.36	(2.19–5.15)
*Period 3*^b^											
NS→NS		44,363	217,093	1,312	604	Reference	—	64	29	Reference	—
FS→FS		10,742	51,494	724	1,406	1.42	(1.26–1.60)	51	99	2.11	(1.33–3.35)
CS→FS	All^c^	3,378	16,014	312	1,948	2.14	(1.86–2.47)	44	275	6.59	(4.38–9.91)
	Illness^d^	1,387	6,428	191	2,971	2.95	(2.48–3.50)	24	373	7.90	(4.55–13.73)
	Others^e^	1,326	6,416	75	1,169	1.43	(1.12–1.83)	12	187	5.04	(2.55–9.94)
CS→CS		13,458	64,696	881	1,362	1.96	(1.75–2.20)	131	202	6.49	(4.08–10.31)

CI, confidence interval; CS, current smoker; FS, former smoker; NS, never smoker.

^a^Period 2 used smoking status from Q00 and Q05, starting at Q05 survey and followed up for five years.

^b^Period 3 used smoking status from Q05 and Q10, starting at Q10 survey and followed up for five years.

^c^All refers to the participants wo quit smoking during two adjacent surveys.

^d^Illness refers to the participants who quit smoking due to illness during two adjacent surveys.

^e^Others refers to the participants who quit smoking due to other reasons during two adjacent surveys.

^f^Adjusted for sex, age, and public health center area.

## DISCUSSION

This population-based prospective study evaluated the impact of smoking status change on mortality in middle-aged Japanese adults. Our principal finding was that relative risk estimates for both all-cause and lung cancer-specific mortality among former smokers were higher in model 2 than in model 1, suggesting that the relative risks for former smokers become higher with updated smoking information. This finding is considered in the context that new quitters were reclassified as former smokers in model 2. However, never or former smokers at baseline who started smoking during the follow-up (never smoker→current smoker and former smoker→current smoker) were reclassified as current smokers in model 2. New quitters in model 2 were generally considered to include those who found quitting easy due to being light smokers or who quit due to life-threatening illnesses. The results for former smokers in model 2 depend on the balance of the two groups. Risks for those who quit due to life-threatening illnesses were indeed high, but we could not determine if the risk for new quitters who had been light smokers was higher or lower than that for persistent former smokers.

Overall, the risk for former smokers become higher in model 2. The relative risk for all-cause mortality among new quitters was significantly higher than that among persistent quitters (Table 4). Among those new quitters who quit due to illness, the relative risk for all-cause mortality was much higher than that for persistent quitters. The increase in relative risk for lung cancer-related death between new quitters and persistent quitters was the same as that for all-cause mortality. Additionally, due to never smokers who started smoking during the follow-up (never smoker→current smoker, *n* = 566; 0.6% of the sample), the crude mortality for never smokers for men, which served as the reference, was overestimated in model 1 because those current smokers were considered never smokers. Therefore, the relative risks among former smokers in model 2 were higher than those in model 1.

Compared to those in model 1, the relative risks for all-cause mortality among current smokers of both sexes were unchanged in model 2 after updating smoking status. First, the relative risks among new quitters who quit due to disease were higher than those for continuous smokers. Second, the relative risks of new quitters who quit for other reasons were lower than those for continuous smokers, except for lung cancer in Period 2. After reclassifying new quitters from current smokers to former smokers in model 2, the relative risk was unaffected by new quitters with both higher risk (current smoker→former smoker with disease) and lower risk (current smoker→former smoker others), which may explain why the relative risks for current smokers in model 2 remained essentially unchanged compared to those for model 1.

Previous studies using both single and multiple surveys often used one approach to the use of smoking information.^5^^,^^7^^,^^10^^–^^14^ Here, smoking status was ascertained more than once for most participants, similar to the British Doctors’ Study, which was initiated in 1951 and followed participants with questionnaires administered in 1957, 1966, 1971, 1978, 1991, and 2001.^14^ The interval between any two surveys was irregular, and the shortest one was 6 years. In contrast, the interval was fixed at 5 years in the present study, shorter than any in the British Doctors’ study. Additionally, the British Doctors’ Study did not compare the impact between the two approaches. Akiba (1994) used data from the Life Span Study (LSS), a large cohort study of atomic bomb survivors in Hiroshima and Nagasaki, and information on smoking habits collected from five sources was consolidated. Risks of lung cancer and cancer in other major sites were evaluated to compare smoking-related risk estimates obtained from two approaches: one with smoking information limited to that available from the first survey (similar to our model 1), and another that incorporated all available information (similar to our model 2).^15^ Compared to Akiba’s results, our estimates for lung cancer risk were much larger based on using updated smoking information among former smokers.^15^ This difference can be explained partially by the fact that the proportion of our subjects whose information was obtained from multiple surveys was 92%, greater than the 42% rate in Akiba’s study. Another possible explanation is that the smoking cessation rate during follow-up in our study was higher than that in Akiba’s study due to the different time periods.

The relative risk for former smokers will decrease further by excluding new high-risk quitters who quit due to illness because the relative risk for new quitters due to illness has a higher value, while that for new quitters due to other reasons has a lower value. The health benefits of smoking cessation could be anticipated, especially for those who quit smoking without disease,^13^^,^^17^ but there remains much to examine to understand the changes in risk occurring after cessation.

Although the present study design was improved using multiple surveys, we could not simulate a case-control study because information on smoking status from a case-control study can be obtained retrospectively, so the correct classification of smoking status can help determine the effects of cessation more accurately. However, we conducted repeated assessments of smoking status in a cohort to mitigate misclassification, and we did not gather information immediately before the events occurred. According to the feasibility of a cohort study, we could not collect information more frequently by decreasing the interval to less than 5 years.

This study has some inherent limitations. First, as with smoking status, the adjustment factors, such as drinking status and body mass index, were also time-dependent. We fixed the adjustment factors during the follow-up for analysis not only to focus on the changes in smoking status but also for technical reasons. Second, we did not adjust for any socioeconomic factor in both models 1 and 2 because there was no information on socioeconomic status available in questionnaires.

### Conclusion

From a large population-based cohort, we clearly demonstrated that the relative risks of all-cause and lung cancer-specific mortality among former smokers become higher using updated smoking status data from repeated surveys during a long-term follow-up. Importantly, smokers who quit due to illness had higher relative risks than did those who continued smoking. The relative risks for all-cause mortality and mortality due to lung cancer among current smokers of both sexes were unchanged using updated smoking status.
